# Impact of textual warnings on emotional brain responses to ultra-processed food products

**DOI:** 10.3389/fnut.2022.895317

**Published:** 2022-11-10

**Authors:** Thayane Ferreira da Costa Fernandes, Naiane Beatriz Ferreira, Rafaela Ramos Campagnoli, Fabio da Silva Gomes, Filipe Braga, Isabel Antunes David, Isabela Lobo

**Affiliations:** ^1^Laboratório de Psicobiologia, Instituto de Ciências Médicas, Universidade Federal do Rio de Janeiro, Macaé, RJ, Brazil; ^2^Instituto de Biodiversidade e Sustentabilidade, Universidade Federal do Rio de Janeiro, Macaé, RJ, Brazil; ^3^Laboratório de Neurofisiologia do Comportamento, Departamento de Fisiologia e Farmacologia, Instituto Biomédico, Universidade Federal Fluminense, Niterói, RJ, Brazil; ^4^Departamento de Neurobiologia, Instituto de Biologia, Universidade Federal Fluminense, Niterói, RJ, Brazil; ^5^Pan American Health Organization, World Health Organization, Washington, DC, United States

**Keywords:** food labeling, consumer behavior, event-related potentials (ERP), EEG, motivation, ultra-processed food, emotion, late positive potential (LPP)

## Abstract

**Background and objectives:**

Ultra-processed food products (UPF) have been associated with numerous non-communicable diseases. Despite this, the addictive nature of UPF, and the aggressive marketing strategies used to promote them, has created a strong emotional connection between UPF and consumers, and supports their increasing UPF global consumption. In view of the emotional link that consumers often have with UPF, modulating emotional reactions to UPF (by using strategies such as textual warnings) is important in changing consumers’ behavior. Since emotions are better understood by assessing individuals’ implicit reactions, we conducted an electroencephalographic study applying the event-related potential technique to investigate whether textual warnings were able to modulate the brain responses to UPF stimuli.

**Materials and methods:**

Twenty-six participants (19 women) viewed pictures of UPF preceded by a warning sentence about the health risks of consuming UPF or a control sentence while the electroencephalogram was recorded. In addition, the participants rated the picture in respect of pleasantness, arousal, and intention to consume. As emotions are associated with motivational circuits in the brain, we focused on a well-known event-related potential brain marker of the motivational relevance associated with emotional stimuli, namely late positive potential (LPP).

**Results:**

The late positive potential amplitude was larger for pictures depicting UPF under the warning condition compared to the control condition, a result that was accompanied by lower pleasantness ratings during the warning condition (compared to the control).

**Conclusion:**

Textual warnings about the negative health consequences of consuming UPF changed the emotional responses toward UPF, possibly increasing the motivation to avoid UPF. These results shed new light on the impact of textual warnings on UPF-evoked emotions.

## Introduction

The relationship between food and emotions has always been of great interest because of the implications for understanding food choices, emotional eating, eating disorders, and marketing strategies of the food industry (1–3). From an evolutionary perspective, foods are emotional stimuli that promote approach behaviors through the activation of a motivational system in the brain named the appetitive motivational system (4). This system ensures that human beings will seize opportunities to obtain food (as for most of human history procuring enough food has been a challenge) and has shaped our approach to sweet, sour, salty, fatty, umami, and starchy foods (5).

However, in the last decades, food environments have changed dramatically, with ultra-processed food (UPF) becoming ubiquitous in the industrialized world (6). In general, UPF are industrial formulations excessive in fats, sodium/salt and/or sugar and depleted or low in micronutrients and other bioactive compounds (7, 8). The food industry designed UPF to be hyper-palatable, which enhances their hedonic value and the pleasure associated with their consumption (9). In addition, the aggressive marketing strategies used by the food industry to promote UPF have long associated UPF with positive emotional content to attract consumers (10, 11). In fact, it has already been observed that viewing pictures of UPF evoke strong emotional reactions in individuals, and may prompt approach behaviors toward UPF that are associated with the activation of the appetitive motivational system (12).

Concerns about UPF and efforts to reduce their consumption are based on a strong body of evidence that links them to the development of obesity and non-communicable diseases, such as cancer and cardiovascular diseases (13–15), and shows them to be socially, economically and environmentally harmful (16–18). Therefore, public policies and actions are essential to achieve an effective reduction in the demand for and offer of UPF. In this context, nutritional warnings, in the form of a front-of-package nutrition labeling scheme, may help consumers to identify products containing excessive amounts of “critical nutrients” (19). This study aims to contribute to the understanding about the brain mechanisms underlying how textual warnings depicting the possible negative health consequences of UPF consumption would discourage consumers from purchasing such food.

Consumers’ purchase decisions are guided by food-evoked emotions (3), so it is vital that nutritional warnings are able to modulate these responses if such public health strategies are to be effective. David et al. (12) collected reports of emotional experiences evoked by pictures of UPF and showed that textual warnings were effective in reducing the strong appetitive motivation evoked by the images and the intention to consume them. Since it can be difficult to capture emotional reactions using words, and given that social desirability bias can be an important confounding factor in experiments that depend on participant’s explicit responses (20), implicit emotional measures represent a valuable tool in food research (21). Indeed, it has been shown that non-verbal food-evoked emotions are better at predicting food choices than verbal food-evoked emotions (2). In this respect, event-related potentials derived from electroencephalographic signals can be used to assess consumers’ implicit emotional motivators. Event-related potentials reflect rapid brain responses associated with food pictures in different experimental conditions (22). Thus, it is possible to obtain valid measures of emotions evoked by UPF pictures and to investigate their modulation by public health strategies such as textual warnings.

Using event-related potentials, it is possible to observe a waveform known as late positive potential (LPP) 300–400 ms after the onset of pictures or words with emotional content (i.e., food stimuli) (23, 24). Any increase in the LPP waveform indicates that the presented stimuli is motivationally relevant for the observer, that is, a larger LPP indicates sustained allocation of attention toward emotionally evocative stimuli (25). The LPP is typically larger for food pictures than objects, and larger for palatable food than less palatable food (22, 26, 27). LPP can also reflect experimental manipulations in food studies (28–31).

The event-related potential approach has already been used to study how cognitive strategies may change emotional responses toward foods (28, 29). However, to the best of our knowledge, no study has used the event-related potential technique to investigate whether textual warnings are able to modulate the powerful influence of UPF (as defined by the NOVA classification system) (7), a food cue that highly activates the appetitive motivational system (12). The present study aimed to add to the findings of the study by David et al. (12), which only used questionnaires to investigate whether textual warnings were able to modulate the emotional brain responses, by also measuring the LPP waveform evoked by UPF. Based on the findings of previous studies that used cognitive strategies, such as inducing the participant into thinking about the long-term consequences of eating unhealthy foods (28, 29), we hypothesized that the LPP would be larger for UPF pictures when preceded by a textual warning compared to those preceded by a control text. In summary, we used the event-related potential technique to gain new insights into the brain mechanisms underlying the effects of textual warnings on emotional processes that drive consumer behaviors in respect of UPF.

## Materials and methods

### Participants

Thirty-three participants, all students at the Federal University of Rio de Janeiro, were recruited. Due to electroencephalogram (EEG) data acquisition problems (e.g., eye blinks, eye movements, muscle activity, and skin potentials), the final sample consisted of 26 volunteers, 19 women and 7 men, aged between 18 and 30 years old [mean (M) = 21.84 y.o., standard deviation (SD) = 2.41]. Sample characteristics are shown in Table 1. All the participants were naive to the purpose of the experiment, were omnivores, and Portuguese native speakers who reported normal or corrected-to-normal vision. The final sample reported no psychiatric or neurological problems. The experiment was reviewed and approved by the Federal University of Rio de Janeiro (*Campus* Macaé) Ethics Committee. The participants provided their written informed consent to participate in this study.

**TABLE 1:** Final sample characteristics.

	Mean	Standard deviation	Min-Max
Age (years old)	21.84	2.41	18–30
Waist circumference (cm)	70.12	9.56	55–94
Body mass index (kg/m^2^)	21.87	3.94	16–33.4

	**n (%)**		

Gender–male	7 (27%)		
Gender–female	19 (73%)		
Monthly household income (up to US$149.80)	5 (19.23%)		
Monthly household income (up to US$374.54)	14 (53.85%)		
Monthly household income (up to US$749.09)	3 (11.54%)		
Monthly household income (up to US$1,123.64)	4 (15.38%)		

Household incomes values refers to Brazilian Real currency (BRL) converted into US dollars (USD) (currency conversion 5.54 BRL: 1 USD).

### Procedure

**FIGURE 1:**
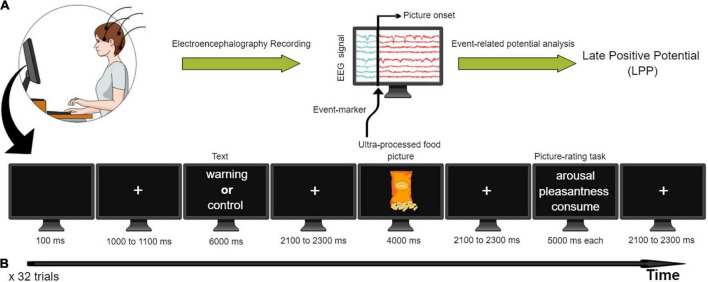
**(A)** The event-related potential waveform late positive potential (LPP) was extracted from the raw electroencephalographic signal. The event of interest (UPF picture) was presented to the participant on a computer screen while the electroencephalographic (EEG) signal was being recorded. Event-markers (i.e., triggers) in the EEG signal time-locked to the UPF picture onset defined the segments from the EEG signal to be analyzed. After conducting 32 trials containing different UPF pictures, the EEG signal was averaged across the segments, revealing the event-related potential waveform–late positive potential (LPP). **(B)** Experimental design showing the sequence of events during the experiment. Participants viewed pictures depicting UPF preceded by a control text (the first block comprising 32 trials, control condition) or a warning text (the second block comprising the 32 remaining trials, warning condition). After viewing the UPF picture, participants rated it in two basic dimensions of emotion: arousal and pleasantness. They also rated their intention to consume the foods depicted in the pictures. UPF, ultra-processed food; EEG, electroencephalographic; LPP, late positive potential.

The experiment was conducted in the mornings in a room with dim ambient light and sound attenuation. The participants sat in front of a computer monitor with their head resting on a forehead/chin supporter approximately 57 cm from the screen. A microcomputer running E-Prime 2.0 (Psychological Software Tools Inc.) timed the presentation of the stimuli (UPF pictures that were presented on the computer screen), delivered the event-markers (triggers) that indicated the onset of the UPF pictures in the electroencephalographic signal, and recorded the keyboard responses. Using the event-related potential technique, the electroencephalographic segments containing the event of interest (the onset of the UPF pictures) were extracted from the electroencephalographic signal and were averaged to produce the event-related potential waveforms (32; see Figure 1A).

Figure 1B describes the sequence of events in each trial. The test started with a black screen (100 ms) followed by a fixation cross shown for 1000–1100 ms (in order for subjects to keep their eyes fixed on screen). Then, a control text (in the first block of 32 trials) or a warning text (in the second block of 32 trials) appeared for 6000 ms. The sentence was replaced by a fixation cross for 2100–2300 ms. After the offset of the fixation cross, a UPF picture appeared in the center of the screen for 4000 ms. The picture was replaced by a fixation cross for 2100–2300 ms. After this, the participants performed a picture-rating task in which: (1) They rated the image according to the emotional dimensions of arousal and valence (pleasantness), by completing the computer version of the Self-Assessment Manikin (SAM) (33); (2) They also rated their intention to consume the UPF depicted in the picture. They used the right-side keyboard numbers to rate the pictures and each rating lasted on the screen for 5000 ms. The SAM arousal dimension indicates the level of emotional arousal, ranging from 1, low arousal, to 9, extremely high arousal. The SAM valence measures the degree of pleasantness of the figure, ranging from 1 for extremely unpleasant to 9 for extremely pleasant. In the intention to consume, the participant estimates how much he/she would like to eat that food shown in the picture, ranging from 1 (none) to 9 (maximum). The above sequence was repeated 32 times (32 trials) for each block (the first 32 trials for the control condition and the following 32 trials for the warning condition, with a short break between them).

The reduced version of hunger scale (34) was used to assess the participants’ subjective hunger before the experimental session, after the control condition, and after the warning condition (see Supplementary Table). The scale consists of reported subjective hunger at the time, and participants were asked to rate first how hungry they were, using a 7-point scale, and then how much of their favorite food they could eat right at the moment using a 6-point scale, with total scores varying from 0 to 11.

### Stimuli

The food depicted in the pictures were classified as ultra-processed based on the NOVA system that considers the extent and purpose of industrial food processing (35). Sixty-four pictures of UPF that are easily found in the Brazilian market were used. “Sugary” product types were represented by 16 pictures of carbonated soft drinks, chocolate bars, chocolate discs and gums; “salty” product types by 16 pictures of corn chips, potato chips, tortilla chips and instant noodles; “saturated fatty” product types by 16 pictures of sausages, salami, nuggets and hot dogs; and “trans-fatty” products by 16 pictures of margarines, filled cookies, wafer cookies, and ice creams. The pictures, as well as the warning and control texts, are the same as those used in David et al. (12), another study by our group, and are shown in detail there (12). The pictures did not include the extrinsic properties of the products, such as the product’s name, brand or package. The set of 32 UPF pictures presented in block 1 (control) in one session for one participant was presented to another participant in block 2 (warning), and vice-versa.

The textual warnings and the control texts were sentences in Portuguese language presented before each UPF in the warning condition and in the control condition, respectively. The warning condition contained sentences addressing the health risk of products, namely the high content of one of the following “components”: sugar, sodium, saturated fat, or trans-fat. For example, a textual warning used for “sugar” pictures was: “*This product contains excessive sugar and, if consumed in large amounts, increases the risk of obesity and dental cavities*.” The control condition contained sized match sentences that also included information about the nutritional contents, for example: “*This product contains sugar and must be kept under refrigeration and be consumed before the expiration date*.” That is, in the control condition, the health risks associated with overconsumption were replaced by the information about the product’s conservation and expiration date (see David et al. (12) for detailed information about the control and warning texts). The control and warning texts were presented in the center of the screen in white font on a black background and occupied approximately 75% of the screen dimensions.

### Electroencephalogram recording and analysis

The EEG signal was recorded using a TiEEG (EMSA Equipamentos Médicos Ltd., Rio de Janeiro, Brazil) recording system with 23 electrodes positioned according to the electrode sites from the 10–20 system: Fpz, Fp1, Fp2, Fz, F7, F3, F4, F8, Cz, C3, C4, T7, T8, Pz, P3, P7, P4, P8, Oz, O1, O2, M1, and M2. All of the electrodes were referenced to Cz during the recording session and re-referenced to the averaged mastoids offline. The sample rate was 600 Hz during data acquisition, and the impedance was kept below 5 kΩ for all of the electrodes. The data were filtered offline using 0.1 Hz high-pass and 30 Hz low-pass digital filters. The offline analysis of the data was performed using the EEGLAB toolbox 14 (36) with MatLab R2016b (MathWorks, Natick, MA, USA). Eye blink artifacts were removed from the data using an independent component analysis (ICA) available in EEGLAB. These components were excluded from the data only after a visual inspection of the topographical maps demonstrated their proximity to the ocular area and established waveform characteristics. The EEG data were epoched from a 200 ms pre-picture onset (baseline period) to a 1000 ms post-UPF picture onset. The event-related potentials waveforms associated with “control” and “warning” conditions were averaged. Epochs containing deviations larger than 100 μV relative to the baseline for any of the electrodes were rejected. The epoch rejection rate did not exceed 20% per subject. The LPP values correspond to mean peak in the window of 400–800 ms after UPF picture onset, which has been usually described for LPP (23).

### Statistical analysis

Mean values of arousal, pleasantness, and intention to consume were analyzed by Student’s paired *t*-tests for comparisons between the control and warning conditions. The effect sizes of the differences were estimated using Cohen’s d values. All data had a normal distribution as assessed by the Shapiro–Wilk W-test (*p* > 0.05 for all variables).

Electroencephalogram data were identified by an analysis of repeated measures ANOVA (using a Greenhouse–Geisser correction when pertinent) with the factors: anterior/posterior (3 levels: central, parietal and occipital electrodes); left/right (3 levels: left, midline and right electrodes), and condition (2 levels: warning and control). Regarding the anterior/posterior factor, central corresponds to the electrodes C3, Cz, and C4, parietal corresponds to the electrodes P3, Pz, and P4, and occipital corresponds to the electrodes O1, Oz, and O2. Although the focus here was on the difference between the LPP for the control and warning conditions (condition factor), it is important to include the other factors to ensure that the waveform included in the analysis presented the pattern expected for LPP. When applicable, Tukey *post-hoc* analyses were performed.

Spearman correlations were performed between LPP measures (LPP warning condition minus LPP control condition) and hunger scores in order to verify if the observed effects were influenced by hunger. The significance level adopted in all analyses was α = 0.05.

## Results

The analysis of participant’s keyboard responses revealed that textual warnings significantly reduced the pleasantness ratings [*t*_(25)_ = −2.83, *p* < 0.01, Cohen’s *d* = 0.37] evoked by the UPF pictures in the warning condition compared with the control condition, in which these pictures were preceded by control texts. That is, subjects judged UPF pictures in the warning condition less pleasant than in the control condition [*M*_(warning)_ = 5.23, SD = 1.29, *M*_(control)_ = 5.67, SD = 1.01]. Neither the subjective measures of arousal ratings [*t*_(25)_ = −1.75, *p* = 0.09, *M*_(warning)_ = 5.44, SD = 1.11, *M*_(control)_ = 5.65, SD = 0.98, Cohen’s *d* = 0.20], or the measures of the intention to consume [*t*_(25)_ = −1.94, *p* = 0.06, *M*_(warning)_ = 4.59, SD = 1.29, *M*_(control)_ = 4.97, SD = 0.92, Cohen’s *d* = 0.34] differed between the warning and control conditions.

**FIGURE 2:**
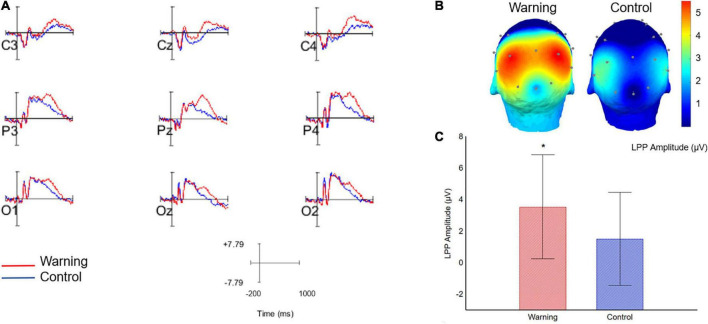
**(A)** Grand averages showing event-related potential waveforms during the warning (red line) and control conditions (blue line) across posterior electrodes (C3, Cz, C4, P3, Pz, P4, O1, Oz, O2). The grand-average ERP plots included the segment during 200 ms pre-UPF picture onset (baseline period) to a 1000 ms post-UPF picture onset. Positive voltages are plotted upward. **(B)** Topographical maps of the scalp (3D head, posterior view) showing the voltage distribution across the posterior electrodes during the warning (left) and control (right) conditions in the time window of 400–800 ms after UPF picture onset. The colormap changes from blue to red, the more positive-going are the waveform deflections over the scalp, measured in micro voltages (μV). Since the LPP is a waveform with positive deflection, it can be observed that it is greater during the warning condition (warm color) than during the control condition. LPP was observed at the posterior electrodes, in agreement with the literature (25). **(C)** Bar chart showing LPP amplitudes during the 400–800 ms time window analysis in the posterior electrodes (voltages were averaged across electrodes) in warning condition (red bar) and control condition (blue bar). *p < 0.05.

In order to illustrate the brain effects related to warning sentences, the grand average waveforms of EEG data for each condition and the topographical scalp maps are presented in Figures 2A,B, respectively. It is possible to observe a remarkably increased amplitude at the posterior electrodes for the warning condition (waves in red) compared to the control condition (waves in blue) (Figure 2A). In fact, the ANOVA showed a main effect of condition [*F*_(1, 25)_ = 11.84, *p* < 0.01], with LPP amplitudes being larger in the warning condition compared to the control condition [*M*_(warning)_ = 3.55 μV; *M*_(control)_ = 1.51 μV], which means a greater emotional reaction in the warning condition than in the control condition (Figure 2C). Compatible with what is expected for the LPP (23), there was also a main effect of anterior/posterior electrodes [*F*_(2, 50)_ = 5.65, *p* < 0.05, ε = 0.67], but this effect did not interact with condition (*p* = 0.17). *Post-hoc* analysis showed that, regardless of warning and control conditions, LPP amplitudes were larger at parietal sites (*M* = 3.74 μV) compared with central sites (*M* = 1.53 μV), and occipital sites (*M* = 2.33 μV) did not differ in LPP amplitude from parietal and central sites. Left/Right factor also reached significance [*F*_(2, 50)_ = 7.81, *p* < 0.01, ε = 0.96] but did not interact with condition (*p* = 0.28). *Post-hoc* analysis showed that, regardless of warning and control conditions, LPP amplitudes were larger at left sites (*M* = 3.02 μV) and right sites (*M* = 2.95 μV) compared to midline sites (*M* = 1.64 μV), with no difference between left and right sites. There were no significant interactions between anterior/posterior and left/right, as well as no triple interaction between factors (all *p*’s > 0.05).

Furthermore, hunger did not affect the observed results since correlation analysis showed no significant results between the EEG data (LPP for Warning—Control across sites) and hunger scores (all *p*’s > 0.05).

## Discussion

The current study used event-related brain potentials to investigate whether textual warnings modulated the emotional brain responses evoked by pictures of UPF. We found a greater brain emotional reaction in the warning condition than in the control condition, manifested as an increase in the LPP waveform. Subjects also rated UPF pictures in the warning condition to be less pleasant than in the control condition. LPP is a well-known brain marker of stimuli motivational significance (25), and the more pronounced LPP combined with the lower feeling of pleasantness evoked by UPF pictures during the warning condition indicate that the textual warnings promoted changes in the participants’ motivational state. Taken together, these results suggest that thinking about the long-term consequences of eating UPF (which was achieved here by applying textual warnings) increases the motivation to avoid them.

A possible explanation to the LPP increase (along with the decrease in the pleasantness ratings) in the warning condition (relative to control) is that textual warnings trigger cognitive strategies that change the perceptions about UPF, modulating the brain emotional responses they evoke. Our results corroborate the study by Meule et al. (28) in which subjects presented a greater LPP when instructed to think about the long-term consequences of eating high caloric food than when they were asked to think about the short-term effects of eating these foods. Similarly, a study by Ma et al. (29) also observed a larger LPP in response to food words when they were paired with names of chronic diseases. It is possible that the LPP increase during the warning condition reflects an increase in emotional states that motivates the individuals to withdraw from stimuli. This interpretation is in line with previous studies that have found an LPP increase in conditions in which food stimuli were not appealing to the participants, such as when food was decomposing (compared with responses to fresh food) (37) or when vegetarians viewed images containing meat (compared to the responses of omnivores) (38). To the best of our knowledge, this is the first study which focused on the LPP evoked specifically by UPF pictures, according to the NOVA classification (7). Most of the previous event-related potential studies used pictures of unhealthy foods, or caloric foods, but those foods were not classified according to the extent and purpose of industrial processing (22, 30). The studies of Lemos et al. (39) and Coricelli et al. (40) did consider UPF, but did not focus on LPP. Currently, the consumption of UPF is a major worldwide public health concern because they are becoming dominant in food environments as they are hyperpalatable, increasingly cheaper, aggressively marketed (16, 41–43) and contain ingredients that drive addictive behavior (9).

It is also important to mention that UPF evokes strong emotional responses that prompt their consumption (12). Our findings are in line with those of David et al. (12) who suggested that textual warnings are able to reduce the appetitive emotional responses evoked by UPF. In the present study, we offer a better understanding on how textual warnings are able to counteract the strong capacity of UPF to attract consumers through the emotions they evoke. In order to develop nutritional warnings that help consumers to resist the appeal of UPF, it is important to consider not only the evidence about *whether* warnings affect consumers’ emotional responses toward UPF but also about *how* and *why* they do this. One advantage of the current study compared to the study by David et al. (12) is that we studied consumer behavior from a brain perspective using EEG, and did not rely solely on applying questionnaires to assess UPF-evoked emotions. Thus, we were able to uncover the brain processes involved in motivational states evoked by UPF and how they are affected by textual warnings. Another advantage is that by applying the event-related potential technique, we provided insights into the brain mechanisms affecting consumers behavior that occur implicitly and are not reported by the participants (44).

The results of the scales showed that there was a reduction in pleasantness in the warning condition compared to the control condition, but no significant changes in the arousal ratings, or intention to consume. Although LPP was previously associated with arousal in the literature (45), more recent studies suggest that LPP represents motivational significance, a phenomenon that is beyond the arousal measured by SAM (25). This is illustrated, for example, in the reactions to pictures with erotic content and exciting sports pictures, both of which are generally rated high in arousal (33). However, erotic pictures evoke a significant LPP, while exciting sports pictures do not (46). Therefore, the larger LPP in the warning condition is reflecting brain processes that cannot be understood simply in the terms of arousal. The absence of a significant effect in intention to consume and arousal is probably due the small sample size for these measures. The mean values for intention to consume (warning = 4.59; control = 4.97) showed a tendency toward a reduction in intention to consume in the warning condition, although the p value did not reach significance level (*p* = 0.06). Furthermore, a reduction in the intention to consume had already been observed in a previous study by our group with the same UPF pictures and same texts, but with a larger sample (12).

Although the block of pictures relating to the warning condition were always shown after those related to the control condition and, consequently, toward the end of the morning, the effect of increased hunger was not able to explain the modulation of LPP in the data. This reveals that the effect of textual warnings can occur regardless of hunger status. This lack of effect in respect of increased hunger may not completely exclude the possibility of an association between the factors, as in other studies LPP has been seen to be greater in response to food cues when participants were hungry compared to satiated (26); however, other studies have not found a direct relationship between hunger and LPP (27, 47).

Some limitations of this study should be highlighted. First, all participants were undergraduate or graduate students, as is common in other studies in this field (28, 29). For this reason, they cannot be considered to be a fully representative sample of the population, as they have a high level of education (incomplete or complete higher education). Second, although the sample size is not small for studies applying the event-related potential technique (48), it is possible that the small size made it difficult to properly observe the effects of individual variability in the sample. There is a final limitation in respect of the control texts, namely that it is possible that the sentences in the control condition were not completely neutral, because the control texts included the name of the macronutrient (i.e., “sugar”). Although the focus in the control condition was to control the health risks associated with overconsumption, the observed differences between conditions may have been even greater if the macronutrient had not been included in the control condition. Further studies should investigate the impact of naming the macronutrients.

In conclusion, textual warnings were able to modulate brain responses related to the motivational significance of ultra-processed food. It is likely that textual warnings increase the activation of motivational brain circuits that facilitate behaviors related to avoiding UPF. These findings build new bridges between the fields of neuroscience and public health, and provide further evidence in respect of the effectiveness of nutritional warnings that we hope will help in respect of public policy efforts to protect the population from the risks of consuming UPF.

## Data availability statement

The datasets analyzed for this study can be found at http://data.mendeley.com/library (49).

## Ethics statement

The studies involving human participants were reviewed and approved by Comitê de Ética em Pesquisa–UFRJ Macaé, Universidade Federal do Rio de Janeiro. The patients/participants provided their written informed consent to participate in this study.

## Author contributions

IL, ID, and FB conceptualized and designed the experiments. TF, NF, and IL collected the data. TF, IL, and ID drafted the manuscript. FG was a staff member of the Pan American Health Organization. All authors contributed to analysis and interpretation, reviewed draft versions of the manuscript for salient intellectual content, provided suggestions and critical feedback, and read and approved the final manuscript.
