# Supplementary material for: Impact of textual warnings on emotional brain responses to ultra-processed food products
Source: Front Nutr. 2022 Nov 10;9:895317. doi: 10.3389/fnut.2022.895317 (PMC9686398; doi:10.3389/fnut.2022.895317)
Supplement: Supplementary file 1 [file Table_1.DOCX]

| **Hunger** | **Mean** | **Standard Deviation** |
| --- | --- | --- |
| Before the experimental session | 4.73 | 2.16 |
| After the control condition | 7.54 | 1.77 |
| After the warning condition | 8.73 | 1.73 |

**Supplementary Table. Hunger scale.**
